# Malnutrition and Its Associated Factors among Rural School Children in Fayoum Governorate, Egypt

**DOI:** 10.1155/2017/4783791

**Published:** 2017-10-23

**Authors:** Wafaa Y. Abdel Wahed, Safaa K. Hassan, Randa Eldessouki

**Affiliations:** Public Health and Community Medicine Department, Faculty of Medicine, Fayoum University, Al Fayoum, Egypt

## Abstract

Malnutrition is an increasing health problem among children in developing countries. We assessed the level of malnutrition and associated factors among school children in a rural setting in Fayoum Governorate, Egypt. A school based cross-sectional survey was conducted on children (6–17 years) in Manshit El Gamal village in Tamia district of Fayoum Governorate. Weight, height, and age data were used to calculate *z*-scores of the three nutritional indicators using WHO anthroPlus. Sociodemographics and lifestyles Data were collected. Prevalence of stunting, underweight, and wasting was 34.2%, 3.4%, and 0.9%, respectively, while obesity was 14.9%. Prevalence of obesity was significantly higher in younger age group of 6–9 years in comparison with older age and was higher in males versus females in 10–13-year-age group. Increasing age, reduced poultry consumption, and escaping breakfast were associated factors for stunting with OR (95% CI) 1.27 (1.17–1.37), 2.19 (1.4–3.4), and 2.3 (1.07–5.03). Younger age and regular employment of the father were factors associated with obesity (OR = 0.753; 0.688–0.824 and OR = 2.217; 1.4–3.5). Malnutrition is highly prevalent in Fayoum in line with the national prevalence and associated with age, gender, regularity of father's employment, and dietary factors.

## 1. Introduction

Infants and young children are the most vulnerable to malnutrition due to their high nutritional requirements for growth and development. The term malnutrition refers not only to deficiency states but also to excess or imbalance in the intake of calories, proteins, and/or other nutrients [1]. In developing countries, malnutrition among children is a major public health concern. It affects all aspects of children's life; its effects are not limited to physical health but extend to mental, social, and spiritual wellbeing [2]. 

The WHO estimates that malnutrition accounts for 54 percent of child mortality worldwide [3], while for children under the age of five years, childhood underweight accounts for 35.0% of all deaths worldwide [4]. In developing countries, 52.0% and 34.0–62.0% of the school-age children are stunted and underweight, respectively [5, 6].

Many factors can cause malnutrition, inadequate food intake, infections, psychosocial deprivation, and insanitary environment as well as lack of hygiene, social inequality, and possibly some genetic contribution. Reports from different organizations like the World Bank documented that children who live in households lacking access to sufficient clean healthy food are more likely to be predisposed to poor nutrition and health related problems than children from food secure households [7].

In Egypt between 1998 and 2004, the prevalence of stunting among schoolchildren remained essentially stable: 14.5% in 1998 and 13.2% in 2004, whereas wasting was 7.8 and 4.1% and underweight was 6.6 and 8.8%, respectively. Overall, malnutrition for this age group does not seem to be improving. Recent studies done by the Egyptian National Nutrition Institute and other research centers revealed that to date malnutrition is a major health concern in the Egyptian community. The problem is affecting different age groups and socioeconomic status [8, 9]. Emam et al. reported that malnutrition disorders affect more than 30% of schoolchildren in Egypt [10]. Malnutrition in rural areas is mostly manifested as underweight while wasting is more common in urban areas [10]. This problem appears to be largely attributable to poor dietary quality and micronutrient deficiencies, such as iron and vitamin A [11].

Even though Fayoum Governorate is one of the large size governorates in Egypt with a population of 3.17 million that is mostly rural (75.0%) and a family size that ranges from 4.1 in urban areas to 4.5 in rural communities [12], there is a significant lack of data on the nutritional status of school children, especially in rural areas. Based on our literature search, only one study has been conducted focusing on a very limited age range for school children (12–15 years) exploring the student's and teacher's attitude concerning obesity and healthy nutritional behavior [13]. In order to formulate a feasible developmental strategy for Fayoum, knowledge on the extent and types of malnutrition is crucial. Lack of such data is a considerable set back in addressing a major health issue and would significantly hinder any efforts towards achieving the declared sustainable developmental goals by 2030.

According to WHO, nutritional status can be assessed through nutritional indicators based on the anthropometric measurements: age, weight, and height due to its sensitivity to the full spectrum of malnutrition. These indicators are as follows: Height-for-age *z*-score (HAZ) (age range: 5–19 years) to measure stunting. Stunting was defined as (HAZ) < −2SD. Weight-for-age *z*-score (WAZ) (age range: 5–10 years) to assess if child is underweight up to 10 years old. After 10 years of age, weight-for-age is not a good indicator where the children grow faster during the period of puberty and can be falsely categorized as excess weight. Underweight is defined as (WAZ) < −2 SD. BMI-for-age *z*-score (BAZ) (age range: 5–19 years): BMI measures weight in kilogram divided by height in meter square. It is a preferred indicator for assessing thinness, overweight, and obesity in children 10–19 years. [14]. Wasting is defined as (BAZ) < −2SD while BAZ > +2SD was defined as obese, for age- and sex-specific *z*-scores, respectively, of National Center for Health Statistics (NCHS) [15, 16].

Our study's aim is to calculate the prevalence of malnutrition using anthropometric measures in school children living in the rural area of Manshit El Gamal village in Tamia district of Fayoum Governorate Egypt, as well as explore the possible sociodemographic and lifestyles factors associated with the condition.

## 2. Methodology

### 2.1. Study Design, Population, and Setting

A cross-sectional school based study was conducted among children aged 6–17, in the rural area: Manshit El Gamal village, in Tamia district of Fayoum Governorate. This rural village was selected purposively, it was chosen because (a) it represents the typical rural areas in Fayoum, with most of its population working in agriculture and its related industry [12]; (b) the village is big enough to include all three levels: primary, preparatory, and secondary schools. The village is the largest of ten villages in this district with a population size of 65000 inhabitants, in a district with a population size of about 425000 based on Egypt population estimate of 2016 [12]. The village has five primary, two preparatory, and one secondary schools with nearly 12000 children enrolled.

### 2.2. Study Subjects, Sampling Size, and Technique

The sample size calculated, using a single proportion formula with maximum allowable error set at 5%, the proportion of malnourished school children at 30.0%, and a 5% significance level, was 322. [6, 17] To account for the design effect of the cluster sample, the sample size needed was doubled to 644. A total 736 school children were recruited to participate in the study.

The students were selected using multistage random technique. First, one school was chosen randomly from the primary and one from preparatory schools in addition to the only high school in the village. Second, in each school, one class per grade was randomly selected. Third, all students in the selected class were included. A total number of 844 students were registered in these classes and 736 participated with response rate 87.2% students. The others were either absent (4.0%) or refused to be included in the study (2.8%). About 6.0% of students, from the young age classes in primary schools, were excluded due to inability of collecting information from them.

### 2.3. Data Collection

Sociodemographics, lifestyles, and dietary habits data were collected through interviews using a structured questionnaire developed based on previous research [5, 7, 10]. The following sociodemographic characteristics were collected: sex, age, parents' education, parent's occupation, number of family members, income, and possessions. Lifestyle and dietary habits that play a role in malnutrition were assessed by asking about frequency of regular exercise, frequency of daily and weekly consumption of red meat, chicken, fish, milk, dairy products, fruit, and vegetables. Even though smoking plays a role in malnutrition we opted not to include it in our results because of related unreliable data. The questionnaire was pretested for accuracy and reliability among 35 students in a nearby school by different interviewers and compared across interviewees and with the student's school record. The questionnaire was modified accordingly.

With the supervision of the researchers, questionnaires were filled by three trained social workers “ra'eedat reefaat” through face-to-face interview during school year 2013/14 over a period of 7 months from October 2014 to April 2015. In each school, the suitable time for collecting data was ensured through an official communication. Respondents were given a clear introduction explaining purpose and objectives of study.

Data on nutritional status of the children was assessed using anthropometric measurements. Weight and height were taken for each student who completed the questionnaire. Weight was measured to the nearest 0.1 kg with an electronic scale, the children were wearing light clothing and without shoes. Child height was measured to the nearest 0.1 cm using a wooden stadiometer placed on a flat surface. All efforts were done to ensure that all questions were completely and accurately answered. Social workers would contact the mothers to complete the missing information related to consumption of fish and other dietary elements especially for younger children.

### 2.4. Ethical Consideration

The study was conducted according to the guidelines laid down for medical research involving human subjects and was approved by Ethics Committee of Faculty of Medicine, Fayoum University. Thorough discussions were undertaken with the school directors regarding the purpose and the contents of the data collection tool, and permission was obtained to conduct the study. Simple explanation about aim of the study was provided to the students to obtain their initial approval. An informed consent including simple explanation about the aim of the study was sent with each student home to be signed by the parent/guardian.

### 2.5. Data Management and Quality

All precautions to ensure quality of the data were performed before, during, and after data collection. Training of data collectors, pretesting of the questionnaire, and standardization of measuring scales of weight and height were undertaken.

Weight, height, and age data were used to calculate *z*-scores of the three different nutritional indicators in comparison to the newly published World Health Organization/National Center for Health Statistics (WHO/NCHS) reference population using the WHO AnthroPlus Software (Version 10.4, 2010) [15, 16].

Malnutrition was assessed according to WHO recommendation using underweight (weight-for-age *z*-score (WAZ) < −2 SD), stunting (height-for-age *z*-score (HAZ) < −2SD), and thinness (low body mass index- (BMI-) for-age < −2 SD overweigh (BMI-for-age ≥ 2SD)) [14].

The socioeconomic score was calculated according to the modified Fahmy and El-sherbini Social Score that includes items included in Table 1. The overall Social Scoring is classified into four levels: less than 15 very low social standards; 15–19 low social standards; 20–24 middle social standards; 25–30 high social standards [18].

Regular physical activity was defined as participation in moderate or vigorous activity for ≥30 minutes/day at least five days per week. Dietary habits were assessed according to the questions regarding daily and weekly consumption of common food groups.

### 2.6. Statistical Analysis

Statistical analyses were performed using the Statistical Package for Social Sciences Version 16.0 for Windows. Means and standard deviations were calculated for bodyweight, stature, and BMI (W/H2) across sex and age groups. The *z*-score of (<−2SD) was calculated to illustrate WAZ, HAZ, and BAZ category of underweight, stunting, and thinness, respectively. Comparison of variables distribution across different categories was done using Chi-square test of significance. Multivariate forward stepwise logistic regression analysis was used to determine associated factors of stunted growth and obesity. These factors were expressed by odds ratio and its 95% confidence interval OR (95% CI); a probability value of type -1 error less of less than 0.05 was considered statistically significant.

## 3. Results

The mean (±SD) age of study group was 12.7 ± 2.4 ranging from 6 years up to 17 years with 58.3% males. Age distribution was similar between male and female participants (*p* = 0.1). More than half of the mothers of the studied sample (67.3%) were illiterate or informally educated, and majority of them were not working (89.4%). Among fathers, nearly 42.0% of them had formal education at or beyond secondary school, and 45.4% had no regular employment. The majority of participants, 55.6%, were members of large family size (more than 5) with half of participants classified in the lower socioeconomic status (Table 2).

The prevalence of stunting (HAZ < −2SD), underweight (WAZ < −2 SD), and wasting (WHZ < −2SD) was 34.2%, 3.4%, and 0.9%, respectively. Prevalence of overnutrition identified in terms of >2SD for HAZ, WAZ, and BAZ was 0.3%, 4.4%, and 14.9%, respectively.

**Figure 1:**
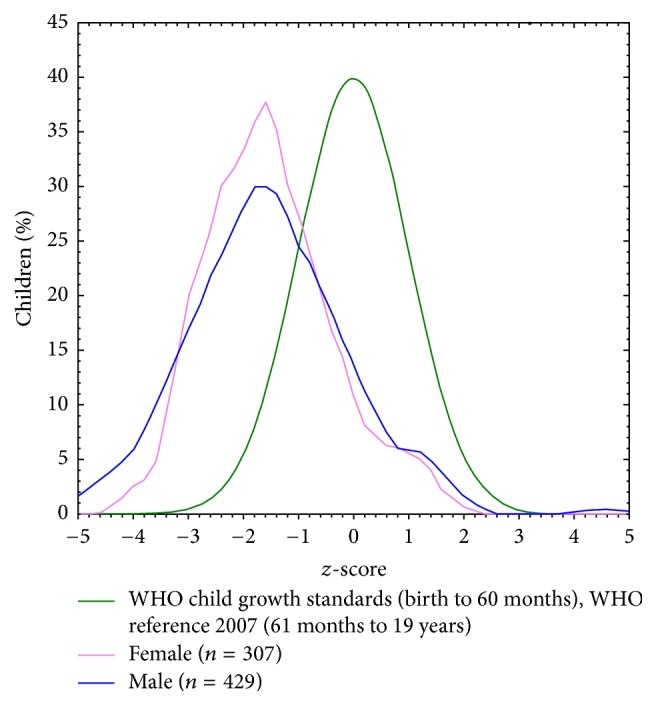
Height-for-age (HAZ) distribution for study group compared to WHO standard reference population.

**Figure 2:**
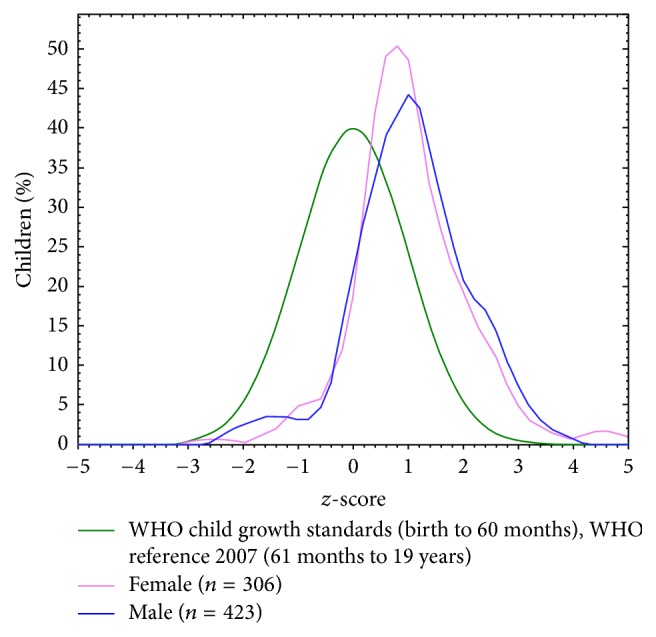
Body mass index-for-age (BAZ) distribution for study group compared to WHO standard reference population.

Double malnutrition problem (overnutrition and undernutrition) identified in our results by high prevalence of both stunting and obesity. The problem of stunting is shown in height-for-age distribution in Figure 1 where the curve is skewed to the left to WHO world standard normal distribution curve. And obesity is seen in BAZ distribution where the curve is slightly skewed to right to WHO world standard normal distribution curve (Figure 2).

The prevalence of stunting was significantly lower in the age group 6–9 (15.3%) than the other two older age groups 10–13 (41.8%) and 14–17 (41.4%) with overall prevalence of 36.2% in females and 32.9% in males. Prevalence of obesity was significantly higher in age group 6–9 (28.3%) than older age 10–13 (12.8%) and 14–17 (4.9%) with overall prevalence of 17.0% in females and 12.1% in males. Significant difference in obesity prevalence between male and female students was reported in the age group 10–13 years *p* < 0.05 (16.7% in males and 8.1% in females) (Table 3).

Assessment of factors associated with stunting and obesity is shown in Tables 4 and 5. In Table 4, stunted growth was significantly higher in absence of mother education, irregular status of father employment, large family size more than or equal to 5. and lower socioeconomic status, while obesity was significantly higher when the father had a higher level of education (secondary or above) and when father had a regular employment.

Table 5 shows that percent of stunted children was significantly higher in participants with poultry consumption less than three times per week 45.9% than participants with three or more poultry times per week consumption 32.2% (*p* = 0.005). In addition, stunting was significantly higher with infrequent fruit consumption 37.8% than daily fruit consumption 28.3% (*p* = 0.008), whereas prevalence of obesity was significantly higher in the category “eating while watching TV” (*p* = 0.008).

Results of the multivariate stepwise logistic regression to study the factors associated with stunting and obesity showed that older age, rare poultry consumption, and escaping breakfast were reported as “stunting” associated factors with OR (95% CI), 1.27 (1.17–1.37), 2.19 (1.4–3.4), and 2.3 (1.07–5.03), respectively. In the same time, daily fruit consumption was a protective factor from stunting with OR 0.614 (0.439–859). For obesity, younger age, male gender, and regular father employment were significant associated factors of obesity with OR 1.553 (1–2.4) and 2.217 (1.4–3.5) (Table 6).

## 4. Discussion

Malnutrition is highly prevalent among children in low and middle income countries. However, wide variations exist in the overall prevalence of underweight, stunting, and wasting among children across countries [19–24]. In a study conducted in governmental school in rural region in India the prevalence of stunting was 32% and underweight was 70% [21]. In Nigeria, the prevalence of stunting was 17.4%, 19.8%, and 16.6%, respectively, among school children [22–24], whereas, in Turkey, only 5.7% of children were stunted [25]. Such variability across different nations can be explained by their social, demographic, economic, nutritional intake, and culture differences between them [24, 26].

The 2014 Egyptian demographic and health survey (EDHS) report showed that chronic malnutrition is continuously increasing since 2000, with a double burden of undernutrition and overnutrition [27].

In the present study, prevalence of stunting, underweight, and wasting among school children in Fayoum was 34.2%, 3.4%, and 0.9%, respectively. Although the prevalence of stunting was high it was less than reported in a survey conducted earlier in Beni-Suef Governorate, where the prevalence of the underweight and stunted was 10.0% and 53.2%, respectively [20]. This difference may be due to the fact that the population studied included both urban and rural areas with different sociodemographic characteristics than what is recorded in our study. However, further investigation is needed to understand the complete picture and map the different economical, nutritional, and social factors affecting nutritional status among Egyptians.

In relation to gender, our findings revealed that the percent of underweight was significantly higher in females than males with no significant difference reported in stunting prevalence. These results are different from the EDHS one, which was conducted on the never-married female and male youth and young adults (10 to 19 years). The EDHS study showed that males (5.0%) were more underweight than females (3.0%) in the age group (10–19 years), with higher prevalence in Upper Egypt and frontier governorates as well as in rural areas [19]. However, Bhargava et al. reported that females were more stunted, underweight, and wasted than males especially in rural schools in India [7]; in Beni-Suef survey it has been concluded that females were more stunted than males (65.3% versus 59.9%) in 10–14-year-age group [20]. This may have an explanation in the cultural preference of boys over girls in rural areas which might translate into a better chance of adequate food.

Stunting was significantly associated with lower level education of mother, irregular employment status of father, large family size, and lower socioeconomic status (Tables 4 and 6), similar to the finding in Sudan and Nigeria [24, 28, 29]. Other factors such as age, sex, birth order of the child, mud floor of the house, religion, and mother's age were reported to be significantly associated with an increased risk of stunting among school children and their parents in Ethiopia [30]. Some of these factors were included in our study but did not show significant association which reflect the variability of the risk factors for malnutrition across different regions.

As the children grow, especially in rural areas they become more active and need more energy, which make them more liable for under nutrition [31]. In Mwaniki and Makokha study, stunting was increased with age and inadequate energy intake [32]. Meat consumption was a protective factor according to Dror and Allen. They reported that eating foods from animal source not only reduced stunting but also improved other anthropometric indices and reduced the morbidity and mortality among undernourished children [33]. Our findings were in line with these statements. In the multivariate analysis increasing age, rare poultry consumption, and escaping breakfast were risk factors while daily fruit consumption was a protective factor from stunting (Table 6).

In addition to undernutrition, Egyptian youth suffers from overnutrition as well with more one-third of the age group (5–19 years) either overweight or obese [34]. In our study, the prevalence of obesity was 14.9% and males tended to be more obese than females in different age groups by using BAZ > +2SD with significant difference especially in the age (10–13 years) (Table 3). The same trend of obesity in males more than females was reported in the 2014 EDHS for the age group (5–14 years); however the tendency was reversed in the report for the age group 15–19 years where females were more overweight and obese [34].

Furthermore, higher prevalence of obesity was positively associated in the age group of 6–9 years with male gender as well as regularity of father's employment which translate into a good socioeconomic status in our results (Table 4). It confirms previous study results which reported a positive association with male gender as well as middle socioeconomic status and highlights that Fayoum Governorate follows the same trend regarding factors of obesity [35–38].

In summary, in Fayoum rural school children, the high prevalence of stunting in girls and the obesity in boys follow the general trend reported in other studies conducted in Egypt and other countries with similar profile (low to middle income) [19–24]. These findings may be explained by the effect of extension of cultural preference for boys over females in Upper Egypt governorates especially in rural areas, as Fayoum is one of the Upper Egypt governorates and mostly rural. The belief in the community is that males are the working group and they are helping their families in earning their living [24, 26].

Double burden of undernutrition and overnutrition is high in Fayoum and is significantly associated with factors such as, age, sex, and reduced animal protein intake, as well as father occupation following the same results in other areas of Egypt as well as other similar countries [24, 28, 29].

Further studies are needed to address factors that showed effect on nutritional status in other regions but were not included in our study due to limited resources as well as the discrepancy between the results of our study and that of the EDHS

## 5. Limitations of the Study

Due to limited resources of this study, one has the following:

- Only rural areas of Fayoum were covered. Different results might be present in urban areas.
- A cluster sample was undertaken with only one rural area selected. Though it is the largest rural areas and has similar composition as other, further study of other rural areas is needed to confirm our findings.
- Despite our effort to reduce confounding factors, some factors such as birth order, religion, and the housing condition that were not studied and accounted for might have affected the results.
- Recall bias may be reported due to subjective nature of the dietary preferences in such children. Moreover as the same questionnaire was used to interview children with a wide age range and it was difficult to interview young children, there is a possibility of information and recall bias of their socioeconomic and lifestyle factors in younger age group.
- Despite high response in our study (87.2%), a higher percentage of subjects under the group age (6–9 y) showed low response rate in comparison with students in other age groups.

## 6. Conclusion

Malnutrition is highly prevalent in rural school children of Fayoum in line with the national prevalence and significantly associated with age, gender, mother's education, and regularity of father's employment. Further studies are needed to address factors that showed effect on nutritional status such as birth order, religion, and mother's age of marriage and confirm our findings.

## Figures and Tables

**Table 1:** The modified Fahmy and El-sherbini Social Score [18].

A	Crowding index (persons per room)	<2 =3, 2− =2, −4 =5
B	Occupation for father or mother	Working = 2 and not working = 1
C	Education for father or mother	Illiterate or read and write = 1, primary = 2, preparatory = 4, secondary = 6, university or higher = 8
D	Family income	Yes and save = 4, yes = 3, sometimes = 2, no = 1
E	Sanitation	All of three (water, electricity, and waste disposal) = 3, two of three = 2, one of three = 1

**Table 2:** Sociodemographic characteristics of study participants (*n* = 736).

Characteristics	Categories	Number (%)
Age in years	6–9	205 (27.9)
	10–13	328 (44.6)
	14–17	203 (27.6)
	Mean ± SD	12.73 ± 2.35

Sex	Male	429 (58.3)
	Females	307 (41.7)

Educational status of mother	Less than secondary education	495 (67.3)
	Secondary and higher education	241 (32.7)

Mother work	Yes	78 (10.6)
	No	658 (89.4)

Educational status of father	Less than secondary education	429 (58.3)
	Secondary and higher education	307 (41.7)

Employment status of father	Regular	401 (54.5)
	Irregular	335 (45.5)

Number of family members	≤5	327 (44.4)
	>5	409 (55.6)

Socioeconomic class of the student^*∗*^	Low and very low	378 (51.4)
	Middle	290 (39.4)
	High	68 (9.2)

^*∗*^Evaluated by modified Fahmy and El-sherbini Score.

**Table 3:** Prevalence of stunting, underweight, wasting, and obesity among study participants by age and sex.

Age groups		HAZ		WAZ^*∗∗*^		BAZ			
		Stunting	*p* value	Underweight	*p* value	Wasting	*p* value	Obesity	*p* value
6–9	M (123)	19 (15.4)	0.874	3 (2.4)	0.346	2 (1.6)	>0.999	37 (30.1)	0.486
	F (82)	12 (14.6)		4 (4.9)		2 (2.4)		21 (25.6)	
	*Total (205)*	*31 (15.1)*		7 (3.4)		4 (2.0)		*58 (28.3)*	

10–13	M (180)	73 (40.6)	0.623			2 (1.1)	*∗∗∗*	30 (16.7)	0.021^*∗*^
	F (148)	64 (43.2)				0		12 (8.1)	
	*Total (328)*	*137 (41.8)*				2 (0.6)		*42 (12.8)*	

14–17	M (176)	49 (38.9)	0.357			0	*∗∗∗*	6 (4.8)	>0.999
	F (148)	35 (45.5)				0		4 (5.2)	
	*Total (315)*	*84 (41.4)*				0		*10 (4.9)*	

*Total *	M (429)	141 (32.9)	0.354			4 (0.9)	>0.999	73 (17.0)	0.063
	F (307)	111 (36.2)				2 (0.7)		37 (12.1)	
	Total (736)	*252 (34.2)*		*6 (0.8)*	*110 (14.9)*				

*p value between age groups*		*<0.001* ^*∗*^				*<0.001* ^*∗*^	

^*∗*^ *p* < 0.05; ^*∗∗*^WAZ is calculated by WHO Anthro PLus up to 10 years; ^*∗∗∗*^invalid chi-square test.

**Table 4:** Association of sociodemographic factors with stunting and obesity among study participants.

		Stunting (252)		Obesity (110)	
		*N* (%)	*p* value	*N* (%)	*p* value
Education status of mother	No (535)	192 (38.8)	<0.001^*∗*^	76 (15.4)	0.656
	Yes (201)	60 (24.9)		34 (14.1)	

Mother work	No (658)	220 (33.4)	0.182	101 (15.3)	0.372
	Yes (78)	32 (41.0)		9 (11.5)	

Education status of father	Less than secondary (427)	144 (33.7)	0.729	50 (11.7)	0.004^*∗*^
	Secondary (309)	108 (35.0)		60 (19.4)	

Employment status of father	Irregular (335)	151 (37.6)	0.037^*∗*^	38 (11.4)	0.013^*∗*^
	Regular (401)	101 (30.2)		72 (17.9)	

Family size	≤5 (327)	99 (30.3)	0.043^*∗*^	57 (17.4)	0.091
	>5 (409)	153 (37.4)		53 (13.0)	

Socioeconomic status	Low and very low (378)	142 (37.6)	0.036^*∗*^	46 (12.2)	0.095
	Middle (290)	95 (32.8)		52 (17.9)	
	High (68)	15 (22.1)		12 (17.6)	

^*∗*^ *p* < 0.05.

**Table 5:** Association of dietary habits and lifestyle factors with stunting and obesity among study participants.

		Stunting (252)		Obesity (110)	
		*N* (%)	*p* value	*N* (%)	*p* value
Eating poultry at least three times per week	Yes (625)	201 (32.2)	0.005^*∗*^	91 (14.6)	0.486
	No (111)	51 (45.9)		19 (17.1)	

Eating fish at least once weekly	No (600)	208 (34.7)	0.930	90 (15.0)	0.608
	Yes (136)	44 (32.4)		20 (14.7)	

Eating milk & dairy product at least once daily	Yes (424)	141 (33.3)	0.512	60 (14.2)	0.481
	No (312)	111 (35.6)		50 (16.0)	

Eating fruits at least once daily	Yes (276)	78 (28.3)	0.008^*∗*^	46 (16.7)	0.310
	No (460)	174 (37.8)		64 (13.9)	

Eating vegetables at least once daily	Yes (253)	80 (31.6)	0.279	46 (18.2)	0.075
	No (483)	172 (35.6)		64 (13.3)	

Regular exercise	Yes	26 (37.1)	0.590	11 (15.7)	0.250
	No	226 (33.9)		99 (14.9)	

Escaping breakfast	Yes (514)	178 (34.6)	0.045^*∗*^	76 (14.7)	0.109
	Sometimes (171)	65 (38.0)		22 (12.9)	
	No (51)	9 (17.6)		12 (29.4)	

Watching TV while eating	Yes (185)	63 (34.1)	0.109	40 (21,6)	0.008^*∗*^
	Sometimes (465)	168 (36.1)		62 (13.3)	
	No (86)	21 (24.4)		8 (9.3)	

^*∗*^ *p* < 0.05.

**Table 6:** Multivariate analysis of factors associated with of stunting and obesity.

Predictors	*p* value	OR (95% CI)
Stunting		
Age	<0.001^*∗*^	1.27 (1.17–1.37)
Poultry at least 3 times per week, yes	<0.001^*∗*^	2.19 (1.4–3.4)
Daily fruit consumption	0.004^*∗*^	0.61 (0.44–0.86)
Escaping breakfast	0.033^*∗*^	2.3 (1.07–5.03)

Obesity		
Age	<0.001^*∗*^	0.75 (0.69–0.82)
Male sex	0.050	1.55 (1–2.4)
Father having a regular employment	0.001^*∗*^	2.22 (1.4–3.5)

^*∗*^ *p* < 0.05.
